# Patient journey through cases of depression from claims database using machine learning algorithms

**DOI:** 10.1371/journal.pone.0247059

**Published:** 2021-02-16

**Authors:** Yoshitake Kitanishi, Masakazu Fujiwara, Bruce Binkowitz

**Affiliations:** 1 Data Science Office, Shionogi & Co. Ltd., Osaka, Japan; 2 Biometrics, Shionogi Inc, Florham Park, NJ, United States of America; University of South Carolina College of Pharmacy, UNITED STATES

## Abstract

Health insurance and acute hospital-based claims have recently become available as real-world data after marketing in Japan and, thus, classification and prediction using the machine learning approach can be applied to them. However, the methodology used for the analysis of real-world data has been hitherto under debate and research on visualizing the patient journey is still inconclusive. So far, to classify diseases based on medical histories and patient demographic background and to predict the patient prognosis for each disease, the correlation structure of real-world data has been estimated by machine learning. Therefore, we applied association analysis to real-world data to consider a combination of disease events as the patient journey for depression diagnoses. However, association analysis makes it difficult to interpret multiple outcome measures simultaneously and comprehensively. To address this issue, we applied the Topological Data Analysis (TDA) Mapper to sequentially interpret multiple indices, thus obtaining a visual classification of the diseases commonly associated with depression. Under this approach, the visual and continuous classification of related diseases may contribute to precision medicine research and can help pharmaceutical companies provide appropriate personalized medical care.

## Introduction

In the pharmaceutical industry, various machine learning methods have been applied to big data for each value chain or across multiple value chains, such as research, development, manufacturing, and post-marketing [1, 2]. As examples, drug discovery target molecules are searched by single nucleotide polymorphisms (SNPs) and genotoxicity is predicted by the quantitative structure-activity relationship (QSAR) in the research phase. Further, the efficacy of drugs is predicted by modeling and simulation of the relationship between pharmacokinetics and pharmacodynamics (PK/PD information) in the development phase, and there are also indirect efficacy comparisons between drugs or signal detections of safety in the post-marketing phase. The information from the research, development, manufacturing, and post-marketing stages is comprehensively integrated for the application of machine learning techniques, for example, for drug repositioning [3, 4]. So far, machine learning has mainly been advanced for improving the efficiency of drug research and development and for determining the differentiation point with other drugs.

However, recent advances in digital technologies have allowed the pharmaceutical industry to deal with new categories of big data [5, 6], for example, from social media networks, wearable devices, smartphone data (application, status of utilization such as tapping and scrolling), disease registries, and claims databases. As their use in clinical trials or other value chain phases can lead to new drug values, we focus on a claims database.

The objectives of a claims database may include understanding treatment practices (daily dose, rates of treatments continuation, drug adherences, post-marketing trends, and patient journeys) and the epidemiology of diseases (onset timing of drug related events, comorbidities, prevalence), as well as exploring the post-marketing safety of drugs (incidence of drug related events). Various studies using claims databases have been carried out from a variety of perspectives, including calculating the incidence of psychosis in ADHD patients [7], investigating the time course of treatment for glaucoma [8], estimating the number of patients with liver disease associated with hepatitis B/C virus infection [9], understanding treatment patterns in patients with hyperlipidemia [10], investigating the prevalence of current treatment practices for rheumatoid arthritis [11], understanding the duration and rate of antipsychotic drugs in outpatients with schizophrenia [12], or exploring the prescribing patterns of antiparkinsonian drugs [13].

However, although machine learning has been used to analyze claims databases, including the identification of subgroups of patients with type 2 diabetes [14] and detecting suicidality in patients with fibromyalgia [15], machine learning should rather be utilized from the viewpoint of the amount and complexity of information contained in claims databases.

Therefore, this paper proposes a novel approach, applying combinations of machine learning methods to claims databases to promote the understanding of a patient’s journey. Specifically, machine learning methods are applied to a claims database to classify a disease by several factors (e.g., medical histories, drugs, background factors) prior to the onset of the disease and the prognoses (e.g., complications) after the onset of the disease. To this end, the Japan Medical Data Center (JMDC) database is used. Association analysis, which is a method of data mining, is used for combination-based disease classification [16–19], as it is efficient for extracting combinations of data that meet certain relevant rules (combinations) from the big data. Additionally, the Topological Data Analysis (TDA) Mapper is a robust and stable method of visualizing data updates, which increases in multidimensional large-scale data by spatially grasping data characteristics [20–23]. As such, we use it to visualize the multi-dimensional indices obtained by association analysis.

To interpret the association analysis results, it is necessary to compare the results of each index and there exist no established methods for understanding such results to date. Therefore, by applying the TDA Mapper to the indices obtained from the association analysis, the relevance of the results can be visually understood. This new approach combines machine learning methods and can help researchers interpret the information and derive deeper insights by visualizing useful information from the claims database.

As one of the diseases for which this new approach is suitable, we focus on the depression which is a typical mental illness as the target disease. The exact cause and prognosis of depression are unclear, and it is reasonable to visualize their diverse relationships by this new approach. Various clinical studies and surveys confirm the usefulness or safety of antidepressant drugs but are carried out after they are marketed. For example, Jick et al. [24] investigated the association between the use of antidepressant drugs and suicidal behavior and reported the possibility of increased risks at the beginning of the treatment, while Weeke et al. [25] investigated the association between antidepressant drug use and out-of-hospital cardiopulmonary arrest and reported how selective serotonin reuptake inhibitors (SSRIs) or tricyclic antidepressant drugs are associated with out-of-hospital cardiopulmonary arrest. The main symptoms of depression could be described on a broad spectrum (low mood, loss of interests, lack of drive, and so on) and the changes in drugs or their concomitant use with other drugs may be appropriate, depending on the symptoms of patients [26]. Therefore, the adverse drug reactions induced by antidepressant drugs after marketing are diverse. These concerns about patient safety for patients with depression have led to several clinical studies and surveys of antidepressants after they have been marketed. Therefore, more safety information should be available prior to prescribing a drug that targets depression, and it is valuable to understand the variety of patient journeys, including whether the drug is likely to work for a patient and whether drug safety will be maintained for a patient based on his/her background.

## Materials and methods

### JMDC database

This study is a retrospective database analysis using the JMDC claims database, which is commercially available in Japan (https://www.jmdc.co.jp/en/index). Since the database consists of unlinkable anonymized data collected for secondary use, ethical approval and informed consent were not required according to Ethical Guidelines for Medical and Health Research Involving Human Subjects by the Ministry of Education, Culture, Sports, Science and Technology, and the Ministry of Health, Labour and Welfare, Japan.

Specifically, the JMDC claims database is an epidemiological receipt database that has accumulated receipt (inpatient, outpatient, dispensing) and medical examination data from multiple health insurance associations since 2005. The cumulative dataset includes approximately 5.6 million subjects (as of June 2018) and it is possible to follow the prevalence rate and incidence for a general population, including healthy individuals. Even if a subject transfers hospitals or uses multiple facilities, the data can still be tracked (https://www.jmdc.co.jp/en/jmdc-claims-database).

We used JMDC data from January 2005 to February 2018 as we started this study from February 2018, and focused on 2-year medical histories (diagnosis information) before the onset of depression as per the F32 (major depressive disorder) code of the International Statistical Classification of Diseases and Related Health Problems (ICD-10) and the 2-year complications after the onset of depression. We extracted data on around 10,000 patients with depression from the JMDC database.

### Association analysis

Medical histories were used to divide the patient journey leading to depression. Association analysis based on the Apriori algorithm which is searching frequent itemset and devising association rules from a database [16–19] was used to extract the combinations of medical histories in the 2 years before the onset of depression from the JMDC database. We used three indices to evaluate the strength of the association between X and Y: support, confidence, and lift, where X and Y are medical histories for the same patients. We used the medical histories 2 years before the onset of depression and did not consider the order or onset time of medical histories.

**Fig 1:**
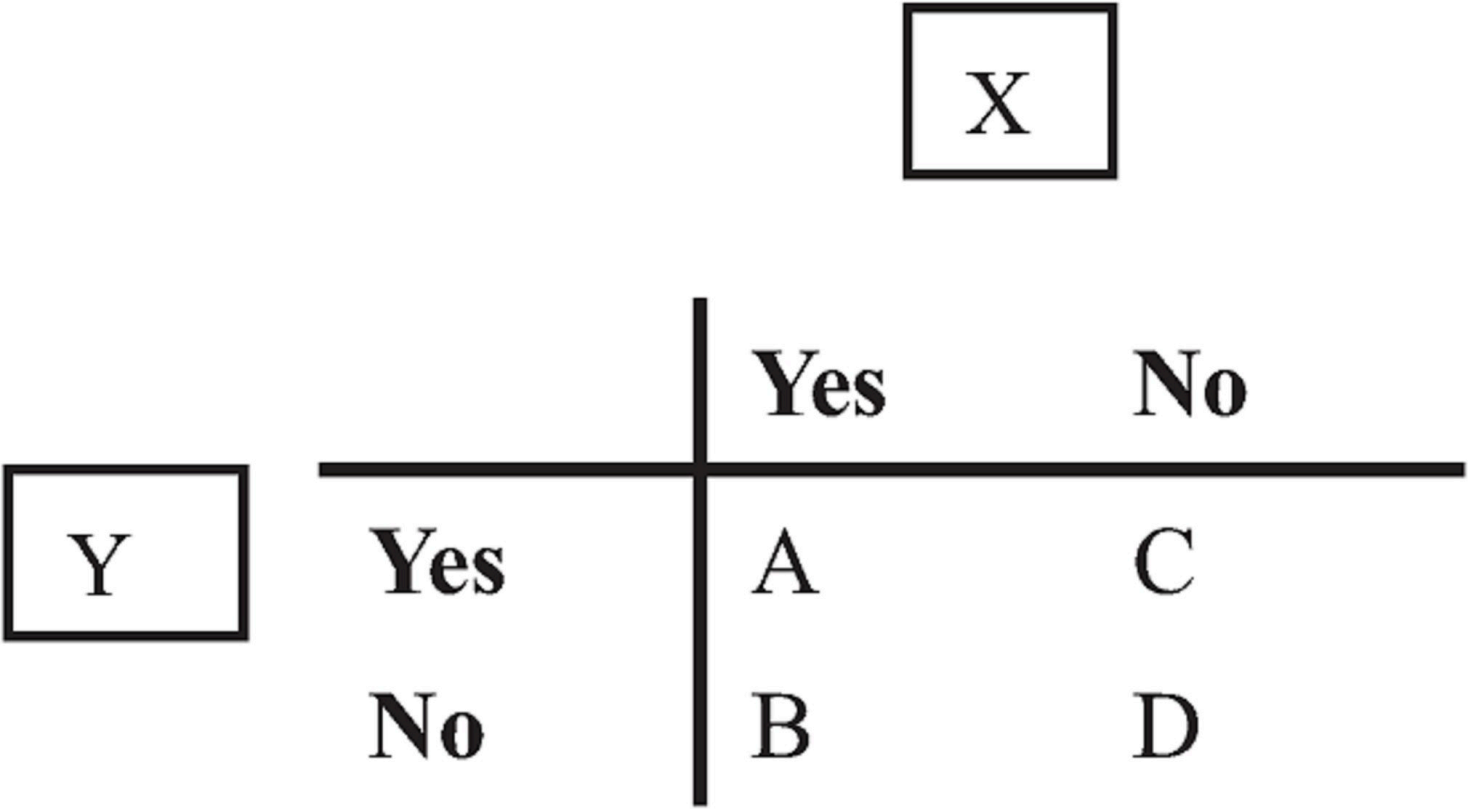
Contingency table.

In the JMDC database, if there exists medical history X for a patient, the variable is set to “Yes,” and if there is no medical history X for a patient, it is set to “No.” X and Y can be represented by a contingency table (Fig 1) for all patients in the JMDC database.

When considering the combinations of medical histories, “A” indicates the number of cases in which both medical histories X and Y are reported as claim data before the onset of depression. Similarly, “B” represents the number of patients with medical history X reported before the onset of depression and no reported medical history Y and “C” represents the number of cases without medical histories X or Y reported before the onset of depression. Finally, “D” represents the number of reported patients without medical histories X or Y before the onset of depression. In the JMDC database, the number of patients reported with depression as F32 (major depressive disorder) is denoted as t. In this case, the support of the combination of X and Y is defined as a/t, confidence as a/(a+b), and lift as {a/(a+b)}/{(a+c)/t}. The lowercase means a realization or an observed value, and the uppercase means an event. These three indices represent the strength of the association between the combination of medical histories X and Y.

Additionally, association analysis using complication data after the onset of depression was also performed for each subset comprising the combinations of medical histories before the onset of the disease. SAS Viya was used for analysis.

### TDA Mapper

The TDA Mapper algorithm makes it easy to visually understand data features, as opposed to the persistent homology that captures the details of data shapes as numerical values. For example, the TDA Mapper is suitable for scenarios wherein features are extracted from big data and comprises four steps: inputting the target data, adjusting the distance function by using the filter function, clustering, and visualizing each parameter.

**Fig 2:**
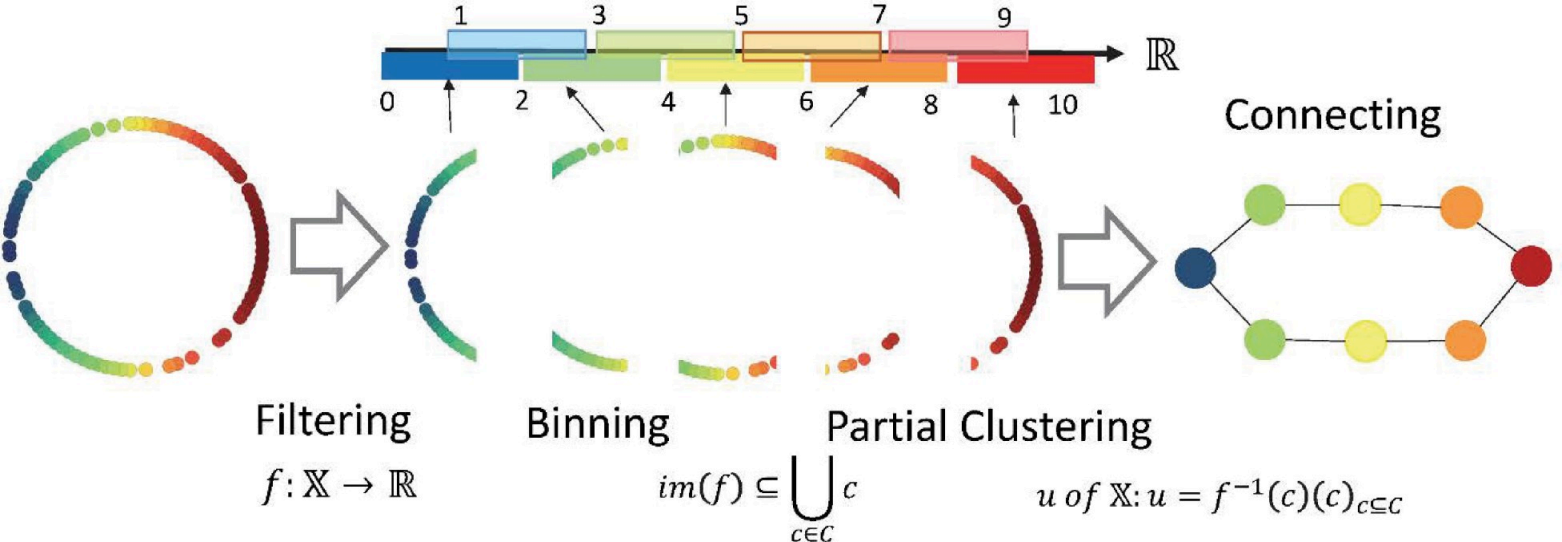
Topological data analysis mapper process. The filtering parameters are as follows: range = 0–10; interval length = 2; intervals = 5; overlap = 50%.

First, the data is inputted in Step 1. The input is N data with M-dimensional features. Next, in Step 2, the distance between any two points in the input data is obtained and an N-by-N distance matrix, X, is created. Using a filter function for this distance matrix, mapping can be performed in a low dimension. The value obtained by mapping in a low dimension is called the filter value. Next, adjust the parameters of the filter function used at this time. In Step 3, the dataset is divided into intervals based on this filter value and clustering is performed at each interval. An overlap is also set when dividing the intervals. By setting the overlap, during visualization, it is possible to draw a line between the clusters with overlapping nodes and show the connections between them. In Step 4, clustering is performed for each interval and a line is drawn between the clusters containing the same nodes to represent the connection. Fig 2 shows the analysis process [20, 27], with specific parameters as an example. Python Mapper [28] was used for the analysis.

## Results

### Association analysis for medical histories

There were 10,188 new depression patients in the JMDC database from January 2005 to February 2018. These patients had a total of 114,691 medical histories in the 2 years before the onset of depression, and 1,051 distinct medical histories. And these patients had a total of 138,549 complications in the 2 years after the onset of depression, and 1,089 distinct complications.

The results of the association analysis for each patient’s medical history data are shown in Table 1. The combinations of medical histories up to the 10th in the descending order of support are shown. The combinations of medical histories with the highest support were X = other anxiety disorders, Y = sleeping disorder, and support (%), confidence (%), and lift were 9.3, 50.1, and 1.1, respectively.

**Table 1:** Association rules for medical histories before the onset of depression.

Rule	Medical history 1	Medical history 2	Support %)	Confidence (%)	Lift
1	Other anxiety disorders	Sleeping disorder	9.3	50.1	1.1
2	Lipoprotein metabolic disorders and other lipemia	Sleeping disorder	8.2	51.8	1.2
3	Other causes of gastroenteritis and colitis, infections and unknown causes	Gastritis and duodenal inflammation	8.2	50.5	1.5
4	Other neurotic disorders	Sleeping disorder	8.2	50.1	1.1
5	Other intestinal dysfunction	Sleeping disorder	7.3	56.8	1.3
6	Gastroesophageal reflux disease	Gastritis and duodenal inflammation	7.2	58.2	1.8
7	Back pain	Gastritis and duodenal inflammation	7.2	50.1	1.5
8	Conjunctivitis	Vasomotor rhinitis and allergic rhinitis	6.1	52.0	2.4
9	Conjunctivitis	Refraction and accommodation disturbance	6.0	50.3	3.0
10	Essential (primary) hypertension (disease)	Sleeping disorder	5.7	56.1	1.3

Among the combinations of medical histories in the top 10 in terms of support, there were five combinations, including sleeping disorder, which was the most frequent. The combination of medical histories without sleeping disorder was X = other causes of gastroenteritis and colitis, infections, and unknown causes; Y = gastritis and duodenal inflammation, with support (%), confidence (%), and lift of 8.2, 50.5, and 1.5, respectively.

**Fig 3:**
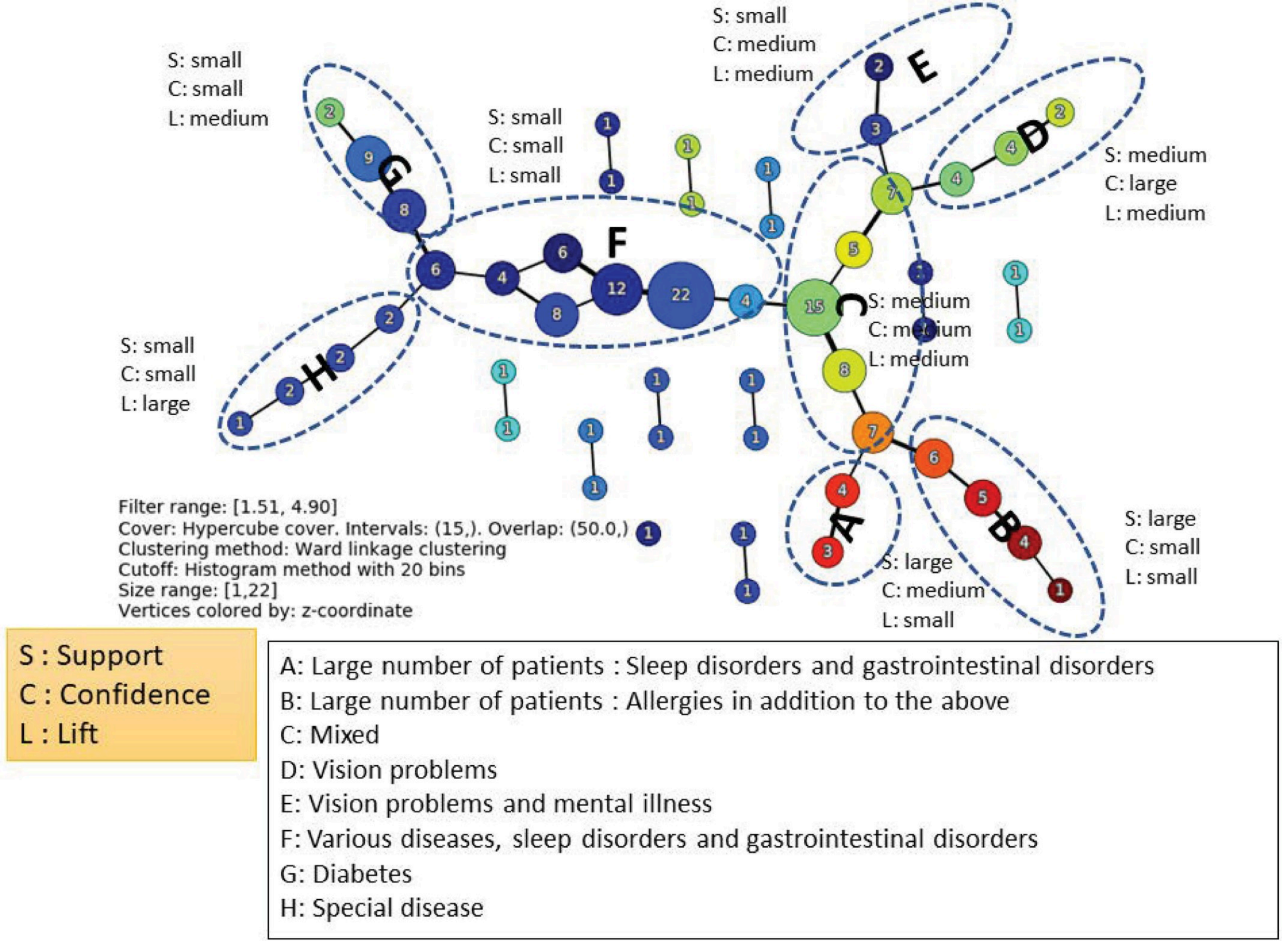
TDA mapper for the association rules of medical histories before the onset of depression.

Additionally, combinations of medical histories with 50 or less occurrences were infrequent and thus excluded, and the TDA Mapper was applied to the values of support (%), confidence (%), and lift for all 109 medical history combinations obtained by applying association analysis. Fig 3 shows the result of applying the Mapper.

The classification was obtained by using support (%), confidence (%), and lift, creating successive combinations of the magnitudes of their respective values. As shown in Fig 3, they can be classified into groups A–H. Looking at the composition of each group, a characteristic medical history is included. The tendency of the magnitude of the three variables in each group is also shown in Fig 3. From these results, it is possible to identify strongly related combinations, even if their frequencies are not high.

### Association analysis for complications

**Fig 4:**
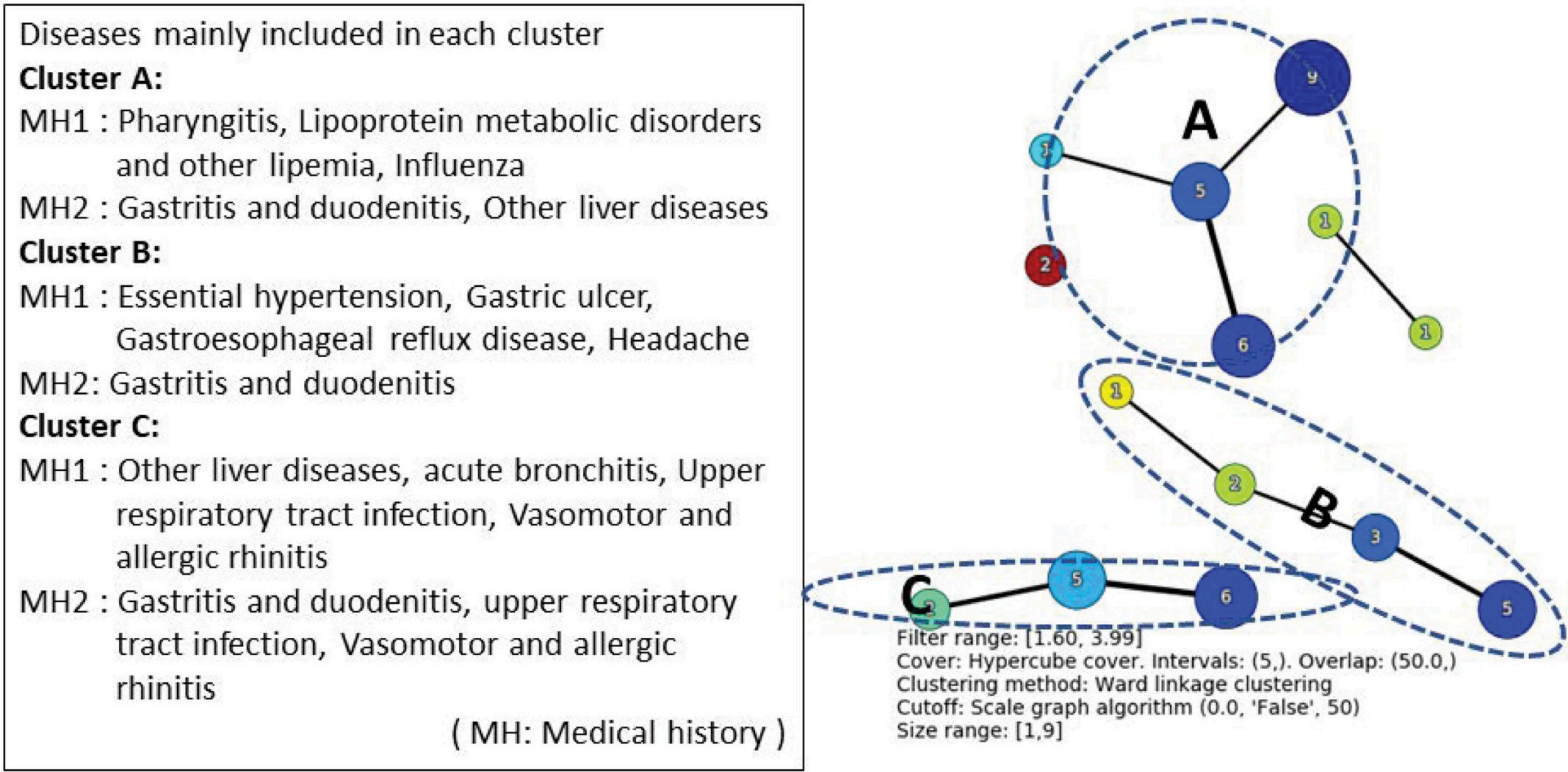
TDA mapper for the result of association rules of complications after the onset of depression for patients with X = other anxiety disorders and Y = sleeping disorder.

By the combinations of medical histories in Table 1, we examined the combinations of complications in the first 2 years after the onset of depression. Among patients with medical histories X = other anxiety disorders and Y = sleeping disorder, which is the most common combination of medical histories for patients with depression, the combination of the most common complications in support was X = multiple and unspecified acute upper respiratory tract infections, Y = gastritis and duodenal inflammation, where support (%), confidence (%), and lift were 16.6, 59.3, and 1.6, respectively (Table 2). Fig 4 shows the results of applying TDA Mapper to the results of the association analysis.

**Table 2:** Association rules for complications after the onset of depression for patients with X = other anxiety disorders and Y = sleeping disorder.

Rule	Medical history 1	Medical history 2	Support (%)	Confidence (%)	Lift
1	Multiple and unspecified acute upper respiratory tract infections	Gastritis and duodenal inflammation	16.6	59.3	1.6
2	Vasomotor rhinitis and allergic rhinitis	Gastritis and duodenal inflammation	15.9	59.5	1.6
3	Multiple and unspecified acute upper respiratory tract infections	Vasomotor rhinitis and allergic rhinitis	14.4	51.4	1.9
4	Vasomotor rhinitis and allergic rhinitis	Multiple and unspecified acute upper respiratory tract infections	14.4	53.9	1.9
5	Other liver diseases	Gastritis and duodenal inflammation	13.8	57.4	1.6

From Fig 4, the classification is approximately carried out for each disease by the index of the three association rules.

Table 3 shows the results of applying association analysis to complications for 2 years in patients with a medical history of the combination with the highest support among the medical history combinations that did not include sleeping disorder (i.e., X = other causes of gastroenteritis and colitis, infections and unknown causes, Y = gastritis and duodenal inflammation). The combination of complications with the highest support was X = keratitis, Y = refraction and accommodation disorders, where support (%), confidence (%), and lift were 4.4, 60.3, and 3.7, respectively. TDA Mapper was not applied in this case because the number of combinations was small.

**Table 3:** Association rules of complications after the onset of depression for patients with X = other causes of gastroenteritis and colitis, infections, and unknown causes and Y = gastritis and duodenal inflammation.

Rule	Medical history 1	Medical history 2	Support (%)	Confidence (%)	Lift
1	Keratitis	Refraction and accommodation disorders	4.4	60.3	3.7
2	Other disorders of the kidney and ureter not elsewhere classified	Other liver diseases	4.3	57.6	3.2
3	Response to severe stress and disability	Sleeping disorder	3.7	52.7	2.0
4	Lacrimal disorders	Refraction and accommodation disorders	2.9	59.0	3.6
5	Purine and pyrimidine metabolism disorders	Other liver diseases	2.5	58.8	3.2

## Discussion

We proposed a new approach for understanding patient journeys by classifying depression through combining medical histories before onset and confirming prognoses such as complications after onset for each medical history type. To this end, we applied machine learning methods (i.e., association analysis and TDA Mapper) to a claims database. This approach leads to a deeper, more diverse, and comprehensive understanding of patient journeys and a better understanding of the patients who should be prescribed such drugs and their prognoses (e.g., occurrence of adverse drug reactions), thus allowing for better personalized patient care. However, the main purpose of this paper is not to provide medical evidence, but to determine the usefulness of analyzing claims database data using machine learning methods. The support of the combinations of medical histories before the onset of depression in Table 1 is slightly lower. That is, the combinations of medical histories were diversified and the rate of each combination was low. The confidence values, which were chosen to be higher to denote a stronger association between X and Y, ranged between 0.5 and 1. Additionally, lift values were greater than 1 in nearly all instances, suggesting a strong association. These data suggest each considered association was moderately strong, although expression rates were low.

Many of the medical histories for which support was superior included medical histories related to gastrointestinal and sleeping disorders. Mental health disturbances have mainly manifested in terms of digestive system and sleep issues, which may have triggered depression [29]. Although these are well known causes of depression, their combination through the claims database is valuable in demonstrating the usefulness of the database.

The results of the association analysis were obtained using several multidimensional indicators, such as support, confidence, and lift. Therefore, it is difficult to intuitively understand the relationship between each combination of indicators. However, by using the TDA Mapper, as in Fig 3, it is possible to visualize relationships. The TDA Mapper can help with two‐dimensional visualization by integrating the three‐dimensional indices of combinations, while maintaining the relationships of the combinations seems to have a good compatibility with the association analyses. Further, the TDA Mapper is more robust than hierarchical clustering because it allows overlap. In other words, the claims database can be interpreted in a robust manner in relation to the changes in the values of the parameters in the association analysis.

Tables 2 and 3 also show that the complications after the onset of depression for patients with medical histories that included sleeping disorders differed substantially from those after the onset of depression in patients with combinations of medical histories that did not include sleeping disorders. Therefore, the complications may differ according to the medical histories before onset and could thus promote a better understanding of patient journeys and lead to better personalized medical care. The data volume in the claims database is expected to increase in the future, but as association analysis is a method for extracting combinations from big data, the results are robust.

We focus on the depression which is a typical mental illness as the target disease. Although the main symptoms of depression could be described on a broad spectrum [26] and there may be the variety of patient journeys for the depression, our approach could detect medical histories and complications from various aspects as shown in Figs 3 and 4. Therefore, our approach seems to have certain applicability to other diseases.

However, this paper is not without limitations. For instance, only the information on medical histories and complications in the claims database was used, but information on backgrounds, drugs, and dates is also available and patient journeys can be better understood by using this additional information. Our approach is also applicable to other diseases and databases including similar information and we would like to validate these results by combining interpretations in clinical practice. The JMDC is a database based on claims received from multiple health insurance societies. Therefore, the percentage of those aged 65 and over is small compared to the actual demographic distribution in Japan and, thus, we cannot deny the possibility that the results are biased in this respect. Additionally, since control patients could not be set, it is not possible to determine whether medical histories and complications were specific to the patients with depression or not. And, we don’t use sequential information on the order and onset time of medical histories, so our method could be developed using this information. This analysis pertains to future work.

## Supporting information

S1 Table (DOCX)
