# Supplementary material for: Patient journey through cases of depression from claims database using machine learning algorithms
Source: PLoS One. 2021 Feb 16;16(2):e0247059. doi: 10.1371/journal.pone.0247059 (PMC7886120; doi:10.1371/journal.pone.0247059)
Supplement: S1 Table — (DOCX) [file pone.0247059.s001.docx]

S1 Table. The overview of JMDC database used in our study.

| **Items** | **Category** | **n (%)** |
| --- | --- | --- |
| Age | n | 10188 |
|  | Mean | 39.8 |
|  | SD | 13.2 |
|  | Min | 2 |
|  | Median | 40 |
|  | Max | 75 |
| Sex | Male | 4576 (44.9%) |
|  | Female | 5612 (55.1%) |
| Medical histories in more than 500 patients | Sleeping disorder | 4429 (43.5%) |
|  | Gastritis and duodenal inflammation | 3240 (31.8%) |
|  | Vasomotor rhinitis and allergic rhinitis | 2179 (21.4%) |
|  | Acute upper respiratory tract infection with multiple or unknown sites | 1945 (19.1%) |
|  | Other anxiety disorders | 1828 (17.9%) |
|  | Refraction and regulation disorders | 1670 (16.4%) |
|  | Acute bronchitis | 1631 (16.0%) |
|  | Other neurotic disorders | 1615 (15.9%) |
|  | Due to other causes of gastroenteritis and colitis, infectious diseases and unknown details | 1604 (15.7%) |
|  | Lipoprotein metabolic disorders and other hyperlipidemia | 1576 (15.5%) |
|  | Diabetes of unknown details | 1513 (14.9%) |
|  | Other liver diseases | 1467 (14.4%) |
|  | Back pain | 1426 (14.0%) |
|  | Other intestinal dysfunction | 1275 (12.5%) |
|  | Gastroesophageal reflux disease | 1229 (12.1%) |
|  | Acute pharyngitis | 1228 (12.1%) |
|  | Gastric ulcer | 1219 (12.0%) |
|  | Conjunctivitis | 1172 (11.5%) |
|  | Other dermatitis | 1143 (11.2%) |
|  | Influenza and influenza virus are not isolated | 1084 (10.6%) |
|  | headache | 1054 (10.3%) |
|  | Essential hypertension | 1004 (9.9%) |
|  | Somatic symptom disorder | 932 (9.1%) |
|  | Asthma | 925 (9.1%) |
|  | Schizophrenia | 865 (8.5%) |
|  | Nausea and vomiting | 850 (8.3%) |
|  | Other hypothyroidism | 773 (7.6%) |
|  | Volume depletion | 731 (7.2%) |
|  | Acute nasopharyngitis | 716 (7.0%) |
|  | Spondylosis | 700 (6.9%) |
|  | Response to severe stress and adjustment disorder | 694 (6.8%) |
|  | Other epidermal thickening | 658 (6.5%) |
|  | Iron deficiency anemia | 654 (6.4%) |
|  | Feeling of dizziness and staggering | 646 (6.3%) |
|  | Acute sinusitis | 625 (6.1%) |
|  | Other spinal disorders, not classified elsewhere | 623 (6.1%) |
|  | Other arrhythmias | 614 (6.0%) |
|  | Other frequent neuropathy | 602 (5.9%) |
|  | Keratitis | 598 (5.9%) |
|  | Chronic sinusitis | 557 (5.5%) |
|  | Migraine | 555 (5.4%) |
|  | Irritable bowel syndrome | 548 (5.4%) |
|  | Acute tonsillitis | 543 (5.3%) |
|  | Bipolar affective disorder | 537 (5.3%) |
|  | Bacterial infection of unknown site | 533 (5.2%) |
|  | Other soft tissue disorders, not classified elsewhere | 520 (5.1%) |
|  | Angina | 514 (5.0%) |
| Complications in more than 500 patients | Sleeping disorder | 4359 (42.8%) |
|  | Gastritis and duodenal inflammation | 3095 (30.4%) |
|  | Acute upper respiratory tract infection with multiple or unknown sites | 2356 (23.1%) |
|  | Vasomotor rhinitis and allergic rhinitis | 2342 (23.0%) |
|  | Acute bronchitis | 2151 (21.1%) |
|  | Refraction and regulation disorders | 1922 (18.9%) |
|  | Diabetes of unknown details | 1901 (18.7%) |
|  | Other liver diseases | 1871 (18.4%) |
|  | Lipoprotein metabolic disorders and other hyperlipidemia | 1778 (17.5%) |
|  | Other anxiety disorders | 1765 (17.3%) |
|  | Due to other causes of gastroenteritis and colitis, infectious diseases and unknown details | 1760 (17.3%) |
|  | Acute pharyngitis | 1676 (16.5%) |
|  | Back pain | 1670 (16.4%) |
|  | Other neurotic disorders | 1590 (15.6%) |
|  | Other dermatitis | 1502 (14.7%) |
|  | Other intestinal dysfunction | 1462 (14.4%) |
|  | Influenza and influenza virus are not isolated | 1453 (14.3%) |
|  | Conjunctivitis | 1406 (13.8%) |
|  | Gastroesophageal reflux disease | 1293 (12.7%) |
|  | Gastric ulcer | 1229 (12.1%) |
|  | Headache | 1190 (11.7%) |
|  | Schizophrenia | 1136 (11.2%) |
|  | Other hypothyroidism | 1111 (10.9%) |
|  | Asthma | 1089 (10.7%) |
|  | Acute nasopharyngitis | 1071 (10.5%) |
|  | Somatic symptom disorder | 1024 (10.1%) |
|  | Nausea and vomiting | 1009 (9.9%) |
|  | Volume depletion | 970 (9.5%) |
|  | Essential hypertension | 899 (8.8%) |
|  | Other arrhythmias | 824 (8.1%) |
|  | Bipolar affective disorder | 802 (7.9%) |
|  | Acute sinusitis | 801 (7.9%) |
|  | Iron deficiency anemia | 793 (7.8%) |
|  | Spondylosis | 786 (7.7%) |
|  | Other epidermal thickening | 778 (7.6%) |
|  | Bacterial infection of unknown site | 750 (7.4%) |
|  | Feeling of dizziness and staggering | 741 (7.3%) |
|  | Keratitis | 740 (7.3%) |
|  | Response to severe stress and adjustment disorder | 733 (7.2%) |
|  | Other frequent neuropathy | 721 (7.1%) |
|  | Chronic sinusitis | 695 (6.8%) |
|  | Acute tonsillitis | 686 (6.7%) |
|  | Other spinal disorders, not classified elsewhere | 652 (6.4%) |
|  | Bronchitis, not explicitly stated as acute or chronic | 650 (6.4%) |
|  | Influenza from which other influenza viruses have been isolated | 649 (6.4%) |
|  | Other anemia | 649 (6.4%) |
|  | Other soft tissue disorders, not classified elsewhere | 647 (6.4%) |
|  | Epilepsy | 641 (6.3%) |
|  | Dermatophytosis | 636 (6.2%) |
|  | Pneumonia, pathogen unknown | 634 (6.2%) |
|  | Cystitis | 630 (6.2%) |
|  | Other disorders of the kidneys and ureters, not classified elsewhere | 628 (6.2%) |
|  | Irritable bowel syndrome | 603 (5.9%) |
|  | Thyrotoxicosis | 602 (5.9%) |
|  | Abdominal pain and pelvic pain | 587 (5.8%) |
|  | Migraine | 583 (5.7%) |
|  | Other disc disorders | 547 (5.4%) |
|  | Disorders of the autonomic nervous system | 545 (5.3%) |
|  | Shoulder injury | 535 (5.3%) |
|  | Angina | 523 (5.1%) |
|  | Lacrimal disorder | 501 (4.9%) |
